# MglA/SspA Complex Interactions Are Modulated by Inorganic Polyphosphate

**DOI:** 10.1371/journal.pone.0076428

**Published:** 2013-10-08

**Authors:** Algevis P. Wrench, Christopher L. Gardner, Sara D. Siegel, Fernando A. Pagliai, Mahsa Malekiha, Claudio F. Gonzalez, Graciela L. Lorca

**Affiliations:** Department of Microbiology and Cell Science, Genetics Institute, Institute of Food and Agricultural Sciences, University of Florida, Gainesville, Florida, United States of America; University of Rochester, United States of America

## Abstract

The transcription factors MglA and SspA of *Francisella tularensis* form a heterodimer complex and interact with the RNA polymerase to regulate the expression of the *Francisella* pathogenicity island (FPI) genes. These genes are essential for this pathogen’s virulence and survival within host cells. Our goal was to determine if an intracellular metabolite modulate these protein/protein interactions. In this study, we identified inorganic polyphosphate (polyP) as a signal molecule that promotes the interaction of MglA and SspA from *F. tularensis* SCHU S4. Analysis of the Mgla/SspA interaction was carried out using a two-hybrid system. The *Escherichia coli* reporter strain contained a deletion on the *ppK-ppX* operon, inhibiting polyP synthesis. The interaction between MglA and SspA was significantly impaired, as was the interaction between the MglA/SspA complex and the regulatory protein, FevR, indicating the stabilizing effect of polyP. In *F. tularensis*, chromatin immune precipitation studies revealed that in the absence of polyP, binding of the MglA/SspA complex to the promoter region of the *pdpD, iglA, fevR and ppK* genes is decreased. Isothermal titration calorimetry (ITC) indicated that polyP binds directly to the MglA/SspA complex with high affinity (K_D_ = 0.3 µM). These observations directly correlated with results obtained from calorimetric scans (DSC), where a strong shift in the mid-transition temperature (Tm) of the MglA/SspA complex was observed in the presence of polyP.

## Introduction

Upon entry into host cells, pathogenic bacteria must evade the attack launched by the immune system, which is directed to eliminate invaders via the oxidative burst. The persistence of microorganisms during periods of unfavorable environmental conditions, including abrupt changes in pH and decreased nutrient availability, is made possible by a series of metabolic changes and the expression of virulence genes that promote survival. The modulation of such expression is predominantly mediated by global transcriptional regulators. The activity of many transcriptional regulators is modulated by inorganic polyphosphate (polyP) and the alarmone ppGpp, thus coupling pathogenesis to the environmental conditions within the host [1], [2].

PolyP is a linear polymer of variable length, linked by high-energy phosphoanhydride bonds [3]. The synthesis of polyP is catalyzed by two polyphosphate kinases (PPK1 and PPK2), both of which are encoded in the genome of most organisms [4]. In *E. coli*, the intracellular level of polyP is controlled by the activity of polyphosphatase (PPX) [5]. The regulatory roles of polyP, in the ability to respond and resist environmental stresses, have been extensively studied in *E. coli* [6]–[8]. PolyP is required for the activation of the *rpoS* gene. In *E. coli,* the sigma factor RpoS (σ^S^) specifically induces ∼50 genes during the transition to stationary phase and under diverse stress conditions [9]. Similar results have been reported in the intracellular pathogens *Shigella flexneri* and *Salmonella enterica* [10]. In these microorganisms, RpoS positively regulates the expression of proteins required to alleviate oxidative stress during host invasion, such as superoxide dismutase, peroxidases, and catalases [11], [12].

The alarmone ppGpp is another molecule that plays an important role during nutritional stress. In *Yersinia pestis*, ppGpp induces the expression of at least three effectors that are injected into the host cell through the type IIII secretion system (T3SS): YopE, YopH, and LcrV. These effectors have been shown to disrupt the host cell cytoskeleton, facilitate the resistance to phagocytosis [13], [14], and trigger interleukin 10 (IL-10) release, suppressing the pro-inflammatory cytokines interferon gamma (IFN-γ) and tumor necrosis factor alpha (TNF-α) [15]. Similarly, after phagocytosis, *S. enterica* serovar Typhi activates the PhoP response regulator, inducing expression of the DNA binding protein SylA, which interacts with ppGpp to induce the expression of genes encoded in the *Salmonella* pathogenicity island 2 (SPI2) [16], [17].

In *F. tularensis*, the importance of ppGpp and polyP in virulence has been documented. Inactivation of the *relA* gene in *F. tularensis* subsp. *tularensis* (*F. tularensis*) SCHU S4 revealed that the absence of ppGpp decreased intramacrophage replication, and virulence in mice was attenuated [18]. At the transcriptional level, Charity *et al*. (2009) [19] were first to establish a link between the presence of ppGpp and the ability of the MglA/SspA complex to interact with FevR [25] (named PigR in *F. tularensis* subsp. *holarctica*, *F. holarctica* [19]) to control the expression of pathogenicity determinants. *In vivo* experiments showed that ppGpp does not affect the mRNA levels of MglA or SspA, nor does it affect the interaction of the MglA/SspA complex with the RNA polymerase (RNAP). It did, however, promote the interaction between the MglA/SspA complex and FevR, as ppGpp is required for FevR activation [19]. The gene encoding polyphosphate kinase (*ppk*) was identified during a screening of *F. tularensis* subsp. *novicida* (*F. novicida*) proteins that were highly expressed in macrophages, but not when grown *in vitro* [20]. Following mutation of the *ppk* gene in *F. novicida* and *F. tularensis,* both strains were defective for intracellular survival and virulence in mice [20]. As a result, polyP is thought to play an important role in *Francisella* pathogenesis, but the mechanism is still unknown.

Here, we determined that the interaction between MglA and SspA is stabilized by polyP. The results obtained provide *in vivo* and *in vitro* evidence for the effector molecule that mediates the interaction of MglA and SspA, which in turn affects the interaction of the complex with other transcription factors.

## Results

### Inorganic Polyphosphate (polyP) Stabilizes MglA and SspA Interactions in *E. coli*

We have recently shown that the interaction between MglA and SspA can be manipulated using small molecules that bind in the cleft region of the heterodimer [21]. The goal of this work was to identify an intracellular metabolite capable of modulating these protein-protein interactions. Charity *et al*. (2009) [19] hypothesized that ppGpp could control expression of MglA/SspA regulated genes. This metabolite is synthesized by the activity of the bifunctional (p)ppGpp synthase/hydrolase, SpoT, and the RelA ppGpp synthase, however, in their experiments with a *F. holarctica* Δ*relA* Δ*spoT* mutant, no effects were observed on the association of the MglA/SspA complex with RNAP. Therefore, we hypothesized that the modifications might be related to the interaction surface directly between MglA and SspA. To test this hypothesis, a new reporter strain for the two-hybrid system was constructed. The *relA* and *spoT* genes were deleted in the AW18 strain (Δ*sspA*) resulting in strain AW20. Strain AW20 was conjugated with KDZifΔZ, to obtain strain AW24, which was then used to determine the interaction of Ft-SspA and Ft-MglA. We found that in a ppGpp null strain, the β-galactosidase levels increased significantly, from ∼2,400 arbitrary units (AU) in the wild type strain (AW23), to 26,000 AU in strain AW24. These results indicate that ppGpp may directly or indirectly act to modulate the physical interaction of MglA and SspA. However, due to the pleiotropic effects associated with the inability to synthesize ppGpp [22], we hypothesized that the accumulation of an intracellular metabolite (i.e., polyphosphate) could also be responsible for the increased interaction of Ft-SspA/Ft-MglA observed in the AW24 strain.

**Figure 1:**
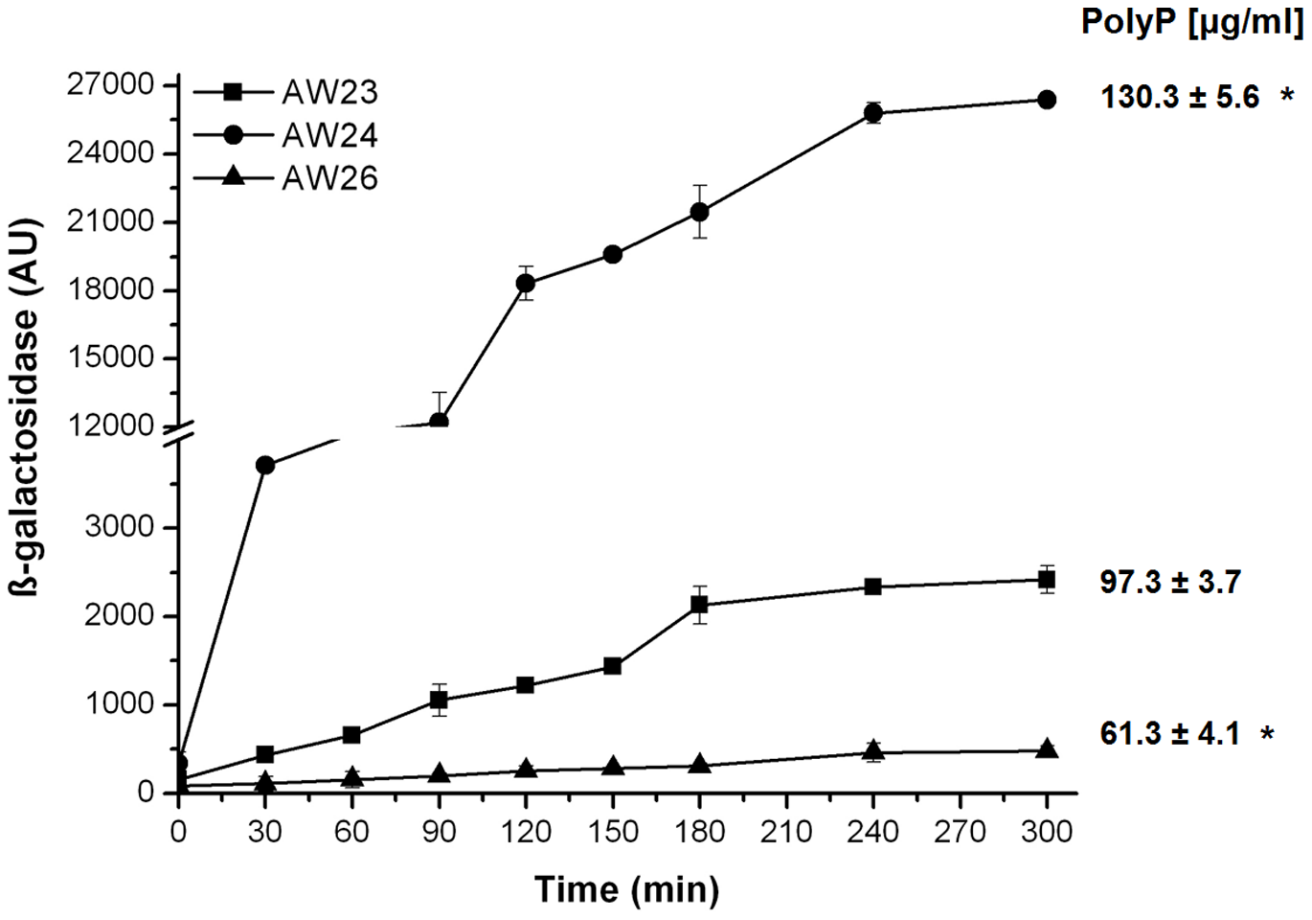
PolyP levels stabilize the Ft-MglA and Ft-SspA interaction in a bacterial two-hybrid system. Transcription activation by the interaction between MglA and SspA fusion proteins from *F. tularensis* SCHU S4 decreases in the absence of polyP. β-galactosidase activity levels from AW23 cells (square), AW24 (circle), and AW26 (triangle). Assays were performed with cells of the *E. coli* reporter strains AW23 (Δ*sspA*), AW24 (Δ*sspA* Δ*relA* Δ*spoT*) and AW26 (Δ*sspA* Δ*ppKppX*) carrying the pBR-*mglA*-ω and pACTR-*sspA*-Zif. Cells were grown and assayed for β-galactosidase activity (expressed in arbitrary units, AU) at different time points. The basal level expression in the AW23, AW24 and AW26 genetic background was similar (Fig. S1). PolyP concentrations were determined as described in the methods section. *p<0.05.

In *E. coli*, polyP is produced by the activity of polyphosphate kinase (PPK) [5]. Recent reports by Li *et al*. [23] and others [7], [24] indicated the essential role of polyP in stress response and stationary phase survival. We hypothesized that the higher β-galactosidase activity observed in the AW24 strain (Δ*relA* Δ*spoT*) was directly related to higher intracellular concentrations of polyP. To test this hypothesis, an *E. coli* strain with a deletion in the *ppK-ppX* operon was constructed (AW21) and conjugated with KDZifΔZ, resulting in strain AW26. The intracellular concentrations of polyP were confirmed using DAPI fluorescence. The concentrations determined were 97.3±3.7 µg/ml in strain AW23, while only 61.3±4.1 µg/ml (p<0.05) were quantified in AW26. The effect of polyP on the interaction of Ft-MglA and Ft-SspA was then followed by β-galactosidase activity. It was observed that in strain AW26, Ft-MglA and Ft-SspA were not able to interact, as evidenced by the reduction of β-galactosidase activity (Fig. 1). Since higher activity was observed in the ppGpp deficient strain (AW24), the level of polyP was determined to investigate its effects in absence of ppGpp. As expected, polyP was found at significantly higher concentrations (130.3±5.6 µg/ml, p<0.05) when compared to the AW23 strain. These results suggest that *in vivo,* high concentrations of polyP are necessary to stabilize the interaction between Ft-MglA and Ft-SspA.

### Effect of PolyP on the Interaction between MglA/SspA and FevR

The putative DNA binding transcription factor FevR was identified in *F. novicida* during a screen of genes requiring MglA and SspA for their expression [25]. FevR physically interacts with the MglA/SspA complex and regulates the same set of genes [19]. However, FevR alone is not sufficient to induce the MglA/SspA regulon [19], [25]. This transcription factor is essential for intramacrophage replication and virulence in the mouse model [19], [25], [26]. Thus, FevR works in parallel with the MglA/SspA complex to activate virulence gene expression.

To study the effect of polyP in the presence of FevR, an *E. coli* bridge two-hybrid system, obtained from Charity *et al*. [19] was used. In this system, the *sspA* gene from *F. holarctica* LVS is provided in a replicative plasmid (pCL-*sspA*), while the *fevR* gene of *F. holarctica* LVS is fused to the Zif protein (pACTR-*fevR*-Zif), and the *mglA* gene of *F. tularensis* SCHU S4 is fused to the ω subunit of the RNAP (pBR-*mglA*-ω). The plasmids were co-transformed into the AW23, AW24, and AW26 reporter strains and the protein-protein interactions were determined *in vivo* by following β-galactosidase activity. No problems were anticipated in the use of heterologous proteins since the MglA, SspA, and FevR proteins from *F. tularensis* SCHU S4 and *F. holarctica* LVS share a 100%, 99% and 100% identity, respectively, at the amino acid level.

**Figure 2:**
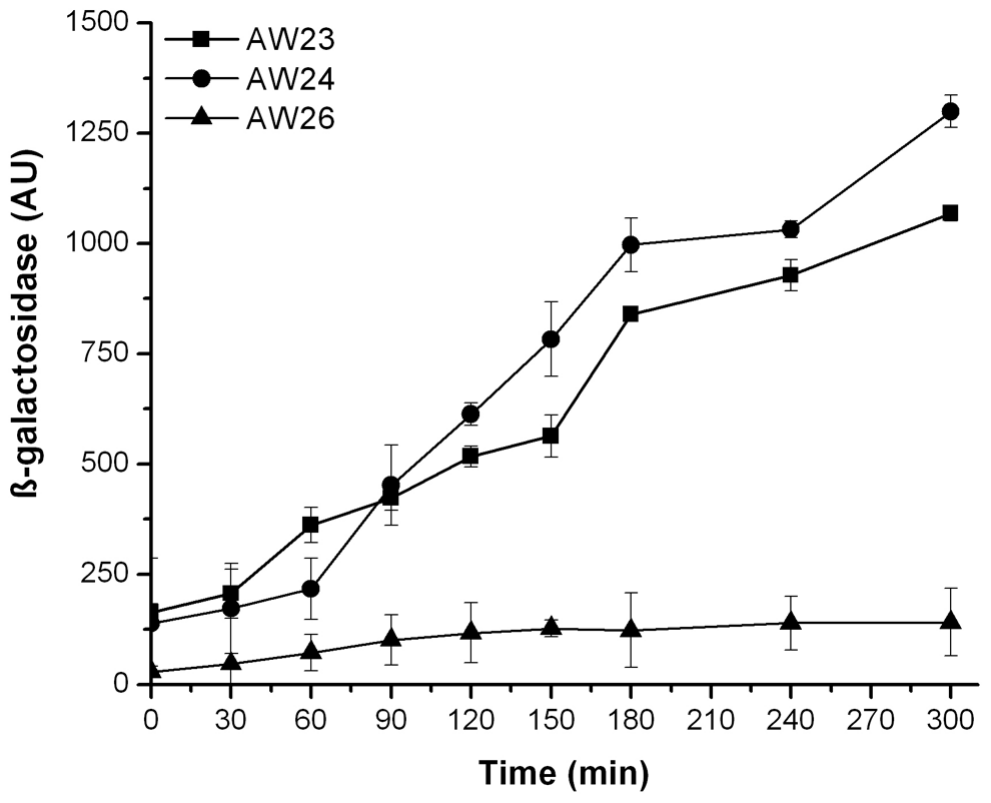
In the absence of polyP, the interaction between FevR, MglA and SspA is impaired. Assays were performed with cells of the *E. coli* reporter strains AW23 (Δ*sspA*, square), AW24 (Δ*sspA* Δ*relA* Δ*spoT*, circle) and AW26 (Δ*sspA* Δ*ppKppX*, triangle) carrying the pBR-*mglA*-ω+pACTR-*fevR*-Zif+pCL-*sspA*. The β-galactosidase activity (expressed in arbitrary units, AU) was determined as described in material and methods. The basal level enzyme activity was subtracted (Fig. S2).

Using these fusions, we were able to reproduce previous observations by Charity *et al.* [19], where the interaction of FevR with the MglA/SspA complex stimulated the transcription of the β-galactosidase reporter gene (Fig. 2). In the AW23 strain, the β-galactosidase activity after 300 min was 1068±17 AU, while in the AW26 strain the β-galactosidase activity was only 141±77 AU (Fig. 2). The AW24 strain showed similar β-galactosidase activity to the AW23 strain (1299±37 AU) in contrast to the large increase observed in this genetic background for the MglA/SspA interactions (Fig. 1). These differences may be explained by the absence of ppGpp described earlier as a putative ligand for FevR (Charity *et al*., 2009). In summary, these results indicate that FevR can interact with the MglA/SspA complex only when polyP is available to stabilize the interaction between the MglA/SspA heterodimer.

### PolyP Stabilizes the MglA and SspA Interaction in *F. novicida*

**Figure 3:**
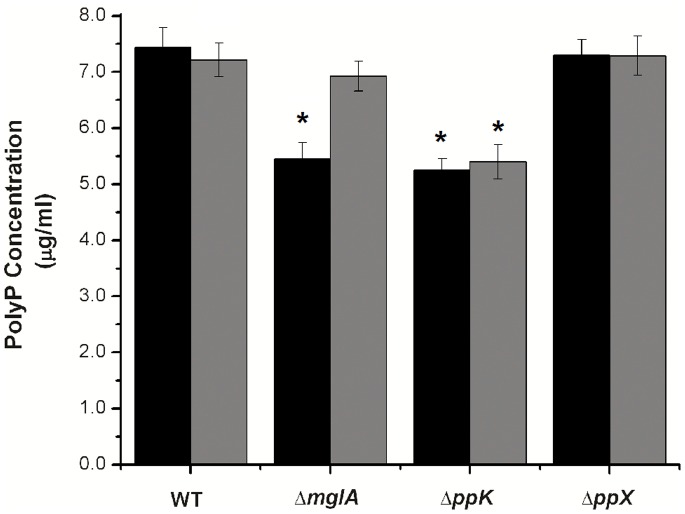
PolyP concentrations in different mutant strains of *F. novicida*. PolyP concentrations were determined in *F. novicida* carrying the empty pKK214 plasmid (black bars) or pSAB (grey bars). PolyP concentrations were determined as described in material and methods. Statistical analyses were performed to compare the polyP concentrations in the wild type strain carrying pKK214, to the mutant strains carrying either pKK214 or pSAB. *p<0.05.

Using the *E. coli* two-hybrid system, we were able to confirm that polyP is required for stability of the MglA/SspA interaction *in vivo*. To validate these observations in *Francisella*, the *F. novicida ppK* mutant strain (FTN_1472) was obtained. The polyP levels of the ppK mutant were compared to the wild type strain, as well as isogenic mutants in *mglA*, *sspA,* and *ppX* (FTN_1414) (Fig. 3). The relative concentrations of polyP were significantly lower in the *ppK* and *mglA* mutants (5.2±0.2 µg/ml and 5.4±0.3 µg/ml, respectively, p<0.05) when compared to the wild type (7.4±0.3 µg/ml), while no changes were observed in the *ppX* mutant (7.3±0.2 µg/ml).

In the *F. novicida* Δ*mglA* strain, decreased polyP production was positively correlated with decreased *ppK* mRNA expression (10 fold decrease), when compared to the wild type strain. These results indicate that *ppK* mRNA expression is dependent on, or is upregulated by, MglA. In the *F. novicida* Δ*sspA* mutant, *a* 3-fold decrease in *ppK* expression was observed. In the absence of MglA or SspA, *ppK* expression levels were similar to other previously identified pathogenicity genes whose expression is dependent on the MglA/SspA complex [21]. Conversely, no changes in the *ppx* mRNA levels were observed in any of the strains tested.

To confirm the dependency of polyP synthesis on the MglA/SspA interaction, we constructed a plasmid to restore the presence of *mglA* or *sspA*. To this end, the *sspA* gene (with its native promoter) and the complete *mglAB* operon (with its native promoter) were cloned in the pKK214 vector (named pSAB). A 6xHis-Tag was introduced on the N-terminus of MglA by site directed mutagenesis. The resulting fusion protein was functional, as it was able to complement the growth defects of a *F. novicida mglA* or *sspA* mutant strain (Fig. S3). The pSAB plasmid was then introduced into the wild type *F. novicida* and mutant strains. As controls, the empty plasmids were also transformed into each genomic background. In the *mglA* mutant strain (containing pSAB) the concentrations of polyP were restored to the levels observed in the wild type, while no changes were observed in the other strains (Fig. 3). These results confirmed the regulatory role of MglA on the synthesis of polyP.

**Figure 4:**
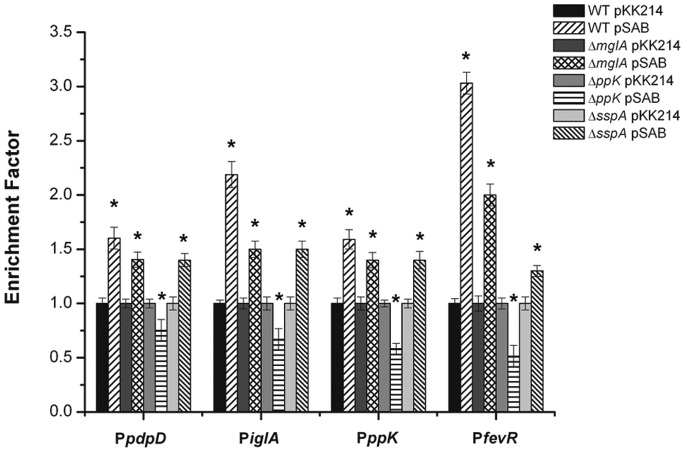
PolyP is required for expression of pathogenicity determinants regulated by the MglA/SspA complex. Chromatin immune precipitation assays were performed with *F. novicida* cells and its mutant derivatives carrying the pSAB. The enrichment factor was calculated as the ratio of amplified DNA (promoter regions of *pdpD, iglA, ppK* and *fevR* genes) in *F. novicida* wild type strain, *mglA*, *ppK* and *sspA* mutants carrying pSAB over the strain carrying the empty pKK214 plasmid. Statistical analyses were performed on the enrichment factor obtained for strains carrying the pSAB over the strain carrying the empty pKK214 plasmid. *p<0.05.

To verify that polyP is required for the expression of pathogenicity determinants mediated by the MglA/SspA heterodimer *in vivo*, chromatin immunoprecipitation (Chr-IP) assays were performed. For these assays, the DNA-binding proteins were cross-linked to their DNA targets *in vivo* with formaldehyde [27]. DNA was extracted, fragmented, and then subjected to Chr-IP with or without the 6xHis-Tag antibody. Crosslinks were then reversed, and the immunoprecipitated DNA was analyzed using quantitative real time PCR. The enrichment factor indicates the extent to which a gene is preferentially precipitated when compared to its abundance in the strain that does not possess the His-MglA. This value was calculated as the ratio of amplified immunoprecipitated DNA in the strain harboring the pSAB plasmid to amplified DNA from the empty pKK214 vector. Ratios of IP DNA pSAB/DNA pKK214 that were over 1.2 were considered significant [27]. As an example of these analyses, we describe the procedure for the *pdpD* gene. Multiple sets of primers were designed in the promoter regions of *pdpD, iglA, fevR*, and *ppK,* and within the open reading frames of *pdpD* and *iglA* (which were used to normalize the values). The enrichment factors were then determined (Fig. 4). The promoter regions of *pdpD, fevR, iglA and ppK* were enriched (between 1.9- and 3.1-fold, p<0.05) in the wild type, the Δ*mglA* and the Δ*sspA* (each carrying pSAB) strains, while no enrichment was observed in the *ppK* mutant strain. These results confirmed that polyP stabilized the interaction of MglA and SspA to promote the transcription of pathogenicity-related genes in *F. novicida*.

### PolyP Binds with High Affinity to the MglA/SspA Complex

**Figure 5:**
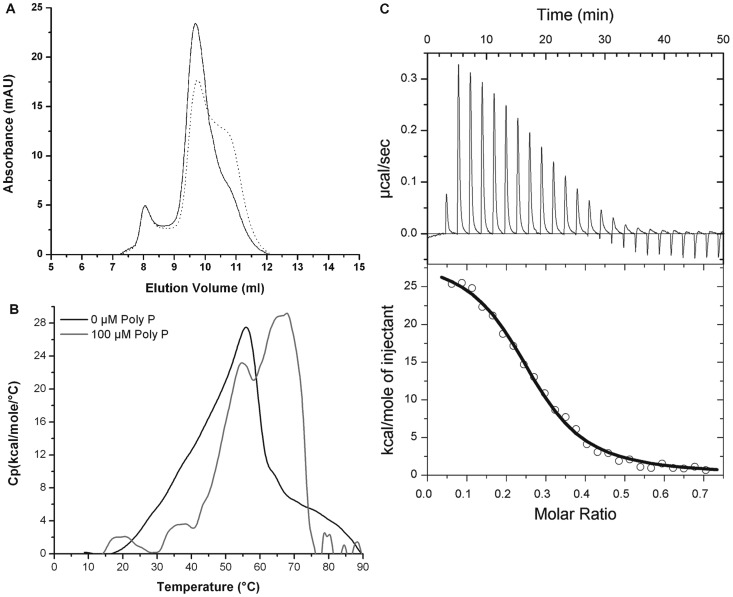
Polyphosphate binds the Ft-MglA/Ft-SspA complex with high affinity. (A) Chromatograms of Ft-MglA/Ft-SspA after the first dialysis cycle (continuous line) or after extensive dialysis (dotted line). (B) Effect of polyphosphate on the thermal unfolding of the Ft-MglA/Ft-SspA complex showing a shift in the transition temperature in the presence of 100 µM of polyphosphate. (C) Isothermal titration calorimetric data for the binding of polyphosphate to the Ft-MglA/Ft-SspA complex. For size exclusion chromatography, 100 µl protein samples in 10 mM Tris (pH 8), 500 mM NaCl were injected onto a prepacked Superose 12 10/300 GL gel filtration column. The DSC experiments were performed in 10 mM phosphate (pH 7.9), 500 mM NaCl in the absence (solid line) or with 100 µM (dashed line) of polyphosphate. For ITC, measurement of heat changes (upper panel) and integrated peak areas (lower panel) of a series of 5 µl injections of 100 µM polyphosphate, into a 16.7 µM protein solution, prepared in 10 mM Tris (pH 8.0), 150 mM NaCl. Experiments were carried out at 18°C.

To determine the effect of polyP on the heterodimer, the oligomeric state was determined by size exclusion chromatography (SEC). As expected, a similar profile of dimers was observed in the chromatograms (Fig. 5A) in both the absence and presence of polyP (100 µM), indicating that further addition of polyP does not affect the dimeric state of the complex. After extensive dialysis, however, we found that a high proportion of the protein was monomeric (Fig. 5A). We hypothesized that during dialysis, the loss of polyP from the complex caused the dissociation of the heterodimer. To test this theory, the concentrations of polyP were determined before and after extensive dialyses. The heterodimer was found to have a polyP concentration of 1595.5±195 ng/ml.mg of protein, which decreased to 679.6±75 ng/ml.mg after the second dialyses. These results indicate that the concentration of polyP was positively correlated to the amount of dimeric MglA/SspA (∼50 kDa) present before and after dialysis.

The thermal unfolding properties of the MglA/SspA complex in the presence of polyP were then established by differential scanning calorimetry (DSC). The calorimetric scans (Fig. 5B) showed that in the presence of 100 µM polyP, the MglA/SspA complex displayed a shift in the transition midpoint temperature from 54.2°C (no polyP) to 66.3°C. These results indicate that polyP binds the MglA/SspA complex and stabilizes the interaction of the heterodimer.

The direct binding of polyP was tested *in vitro* by isothermal titration calorimetry (ITC). It was determined that polyP binds with high affinity (*K_D_* = 0.36±0.03 µM) to the MglA/SspA complex (Fig. 5C). The binding of polyP was an exergonic reaction (Δ*G* = −4482.2 kcal/mol) driven by favorable entropy changes (*T*Δ*S* = 4512.2 kcal/mol) and unfavorable enthalpy changes (Δ*H* = 29.7 kcal/mol). Additional phosphate compounds, including NaPO_4_, pyrophosphate, and triphosphate, were also examined by ITC and DSC, but did not bind to the MglA/SspA complex (data not shown).

## Discussion

In this report, we identified a native molecule (polyP) that acts as mediator of the molecular interactions in the MglA/SspA complex, the main factor in triggering the expression of pathogenicity determinants in *F. tularensis*. The biological role of polyP presented here is supported by a previous *in vivo* report by Richards *et al*. [20]. Their studies revealed that mutations in FTN_1472/FTT_1564, a region encoding a putative polyP kinase (PPK2), resulted in impaired intracellular growth and virulence. This phenotype was correlated with lower intracellular polyP concentrations [20].

PolyP is synthesized as a linear polymer of variable length by the activity of polyphosphate kinases. The activity of these enzymes has been extensively characterized and divided in two classes, PPK1 and PPK2. The PPK1 enzyme shows a preference for polyP synthesis using ATP as a substrate, while PPK2 generally uses polyP for the phosphorylation of GDP to GTP [28]–[30]. The presence of both, PPK1 and PPK2 has been detected in the genomes of diverse microorganism [4]. In contrast, *in silico* analyses revealed few microorganisms (i.e., *Corynebacterium glutamicum*) encode for only the PPK2 homolog [4]. Analysis of the *Francisella* genomes revealed the presence of PPK2 homologs; however, no PPK1 homologs were identified. Interestingly, the *C. glutamicum* PPK2 (NCgl2620) enzyme was found to synthesize polyP from ATP or GTP, in contrast to the PPK2 of *Pseudomonas aeruginosa*, which was found to have NDP kinase activity [29], [31]. Of note is the fact that *P. aeruginosa* uses PPK1 for the synthesis of polyP. We found that *F. novicida* mutants defective in PPK2 (FTN_1472) had significantly lower concentrations of polyP, in agreement with previous *in vivo* results reported in *C. glutamicum* deficient in PPK2. These observations indicate that the PPK2 enzyme catalyzes the synthesis of polyP in *F. tularensis*. Interestingly, we also observed that the polyP levels were decreased in the *F. novicida mglA* and *sspA* mutant strains, thus establishing a link between the physiological status of the cell and the expression of pathogenicity determinants.

In *E. coli*, it was observed that while the levels and activity of PPK1 and the exo-polyphosphatase PPX remain constant throughout the growth curve, the intracellular levels of polyP are high during exponential phase, and decline during the stationary phase of growth. These observations were positively correlated with high ppGpp concentrations at the latter growth phase. Extensive *in vitro* and *in vivo* data showed that the activity of PPX is allosterically modulated by the concentrations of ppGpp [5]. In *F. tularensis*, Charity *et al*. [19] established a link between the synthesis of the stringent response alarmone, ppGpp, and the ability of the MglA/SspA complex to interact with FevR to control the expression of pathogenicity determinants. The two-hybrid system described by Charity *et al*. [32] was used to assess the direct effect of ppGpp on the MglA/SspA interaction. The *E. coli spoT* and *relA* genes were inactivated to create a ppGpp null reporter strain, where MglA/SspA interactions were observed to increase. These puzzling results were correlated with increased concentrations of polyP. Previous studies in *E. coli* [24] have shown that a *relA spoT* double mutant did not accumulate polyP when tested in MOPS minimal media [5]. This apparent discrepancy could be explained by the different growth conditions used in the two approaches (complex LB media versus minimal MOPS minimal media), which can be a fundamental deciding factor for degradation of the polyP polymer. Based on previous reports and our polyP determination in *Francisella*, it is possible that PPX (FTN_1414) is regulated by a similar allosteric mechanism dependent of ppGpp. We propose that in *Francisella,* the fluctuation in ppGpp levels would affect the concentrations of polyP, which in turn will affect the stability of the MglA/SspA complex and their ability to induce stress responses and/or the expression of pathogenicity determinants.

PolyP is a ubiquitous compound, with diverse signaling functions. In *E. coli* it has been associated with stationary phase survival, nutrient and stress responses, as well as biofilm formation [33]–[35]. Recent implications of polyP as a modulator of pathogenic traits have been established for several bacterial pathogens, including: *Shigella flexneri, Salmonella enterica* [10], *Campylobacter jejuni* [36], *Pseudomonas aeruginosa* [37], [38] and *Francisella tularensis* [20]. In *F. novicida* mutations in FTN_1472 (PPK2) or MglA, resulted in impaired intracellular growth and virulence [20]. In this report, we present direct evidence that polyP binds to the Ft-MglA/Ft-SspA complex with high affinity (K_D_ = 0.3 µM). These K_D_ values are comparable to enzymes that bind polyP with a very low *K_m,_* such as the polyP glucokinase (*K_m_* = 2.9 to 5 µM) [39]. The affinity values obtained for the MglA/SspA complex are biologically relevant, since the concentrations of polyP required for stress survival in *E. coli are* as low as 0.1 mM [34]. The polyP accumulated during stationary phase, however, can reach as high as 50 mM [40].

It has been shown that MglA also modulates the expression of a large set of genes involved in general stress response and stationary phase survival [41]. *F. tularensis* strains deficient in MglA synthesis are sensitive to oxidative stress conditions, a phenotype that has been determined for PPK mutants of other microorganisms [41]–[43]. In *F. novicida* we determined a direct correlation between PolyP synthesis and binding of the MglA/SspA complex to the promoter regions in FPI genes. Most interestingly, we determined that expression of the *ppk2* gene is downregulated in *mglA* mutants, an observation that was correlated with lower concentrations of polyP. Thus, the transcriptional regulation of *ppk2* by MglA may explain, at the molecular level, the lower tolerance to stress conditions observed in *mglA* mutants [41].

The role of polyP as a signal molecule has been studied extensively in *E. coli* [34], [44]. The direct binding of polyP to proteins, however, was only observed in a few cases, such as the Lon protease in *E. coli* [45], and σ^80^ from *Helicobacter pylori* [2]. Based on our size exclusion chromatography data, we hypothesize that the binding site of polyP is located within the heterodimer interface of the MglA/SspA complex. Preliminary structural modeling revealed that the interface does not contain a patch of lysines (19 residues over 59 residues) as previously determined to be the polyP binding site in σ^80^ [2], however, the interface surface area does contain several positively charged and hydrophobic residues. Further experiments at the structural level are being performed to test this hypothesis.

## Experimental Procedures

### Bacterial Strains and Growth Conditions

The bacterial strains and plasmids used in this study are listed in Table 1. *F. novicida* U112 and its mutant derivatives (obtained from *Francisella tularensis* subsp. *novicida*, “Two-Allele” Transposon Mutant Library, Plates 1–33, NR-8034) were routinely cultured at 37°C with aeration, in modified tryptic soy broth (TSB) (Difco Laboratories, Detroit, MI) containing 135 µg/ml ferric pyrophosphate and 0.1% cysteine hydrochloride. For CFU enumeration, cysteine heart agar medium (Difco) supplemented with 1% hemoglobin solution (BD Diagnostics, Sparks, MD) (chocolate II agar plates-CHOC II) was used. *E. coli* strains were grown at 37°C under aerobic conditions in Luria-Bertani medium (LB) (Difco) or on LB agar plates. *E. coli* strains DH5α (Invitrogen, Carlslab, CA), XL1-Blue, and JM109 (Stratagene, La Jolla, CA) were used to propagate the plasmids for protein purification, point mutations, and two-hybrid systems, respectively. *E. coli* strain BL21-Rosetta(DE3) (Novagen, Gibbstown, NJ) was used to co-express Ft-MglA and Ft-SspA. When required, the medium was supplemented with ampicillin (100 µg/ml), tetracycline (10 µg/ml), kanamycin (50 µg/ml), or streptomycin (50 µg/ml). All antibiotics and chemicals were purchased from Sigma (St. Louis, MO).

**Table 1:** Bacterial strains, and plasmids used in this study.

Strain, or plasmid	Genotype, or description	Reference, or source
**Strains**		
*F. novicida*		
Wild-type (WT)	*F. novicida* U112 strain	[54]
WT-pKK214	WT carrying pKK214	This work
WT-pSAB	WT carrying pSAB	This work
Δ*mglA*	tnfn1_pw060419p04q129; Km^r^	BEI Resources
Δ*mglA*-pKK214	Δ*mglA* carrying pKK214	This work
Δ*mglA*-pSAB	Δ*mglA* carrying pSAB	This work
Δ*sspA*	tnfn1_pw060323p02q162; Km^r^	BEI Resources
Δ*sspA*-pKK214	Δ*sspA* carrying pKK214	This work
Δ*sspA*-pSAB	Δ*sspA* carrying pSAB	This work
Δ*ppK*	tnfn1_pw060418p01q136; Km^r^	BEI Resources
Δ*ppK*-pKK214	Δ*ppK* carrying pKK214	This work
Δ*ppK*-pSAB	Δ*ppK* carrying pSAB	This work
Δ*ppX*	tnfn1_pw060323p05q104; Km^r^	BEI Resources
Δ*ppX*-pKK214	Δ*ppX* carrying pKK214	This work
Δ*ppX*-pSAB	Δ*ppX* carrying pSAB	This work
Δ*fevR*	WT *FTN_0480::Km*	[25]
Δ*fevR*-pKK214	Δ*fevR* carrying pKK214	This work
Δ*fevR*-pSAB	Δ*fevR* carrying pSAB	This work
*E. coli*		
DH5α	F^–^ Φ80*lac*ZΔM15 Δ(*lac*ZYA-*arg*F) U169 *rec*A1 *end*A1 *hsd*R17 (r_K_^–^, m_K_^+^) *pho*A*sup*E44 λ– *thi*-1 *gyr*A96 *rel*A1	Invitrogen
BL21-Rosetta(DE3)	F^–^ *ompT hsdS*_B_(r_B_^–^ m_B_^–^) *gal dcm* (DE3) pRARE	Novagen
Ft-MglA/SspA	BL21-Rosetta(DE3) carrying p15TV-*mglA* and pCDF-*sspA*; Amp^r^, Str^r^	[21]
XL1-Blue	*recA1 endA1 gyrA96 thi-1 hsdR17 supE44 relA1 lac*[F *proAB lacI* ^q^ *Z*Δ*M15* Tn*10* (Tet^r^)]	Stratagene
JM109	e14^–^(McrA^–^) *recA1 endA1 gyrA96 thi-1 hsdR17* (*r*_K_^–^ *m*_K_^+^) *supE44 relA1* Δ(*lac-proAB*)[F *traD36 proAB lacI* ^q^ *Z*Δ*M15*]	Stratagene
FW102	[F^−/^ *araD(gpt-lac)5* (*rpsl*::Str )]; Str^r^	[46]
AW18	[F^−/^ *araD(gpt-lac)5* (*rpsl*::Str ) Δ*sspA*]; Str^r^	[21]
AW19	[F^−/^ *araD(gpt-lac)5* (*rpsl*::Str ) Δ*sspA* Δ*relA*]; Str^r^	This work
AW20	[F^−/^ *araD(gpt-lac)5* (*rpsl*::Str ) Δ*sspA* Δ*relA* Δ*spoT*]; Str^r^	This work
AW21	[F^−/^ *araD(gpt-lac)5* (*rpsl*::Str ) Δ*sspA* Δ*ppKppX*]; Str^r^	This work
KDZifΔZ	[F’*lacproA+,B+(lacI^q^ lacPL8)/araD(gpt-lac)5* (D*spoS3*::cat )]; Km^r^	[32]
AW23	AW18 conjugated with KDZifΔZ; Str^r^, Km^r^	[21]
AW23-1	AW23 carrying pBR-*mglA*-ω and pACTR-*sspA*-Zif	[21]
AW23-2	AW23 carrying pBR-GP-ω and pACTR-AP-Zif	[21]
AW23-12	AW23 carrying pBR-*mglA*-ω, pACTR-*fevR*-Zif and pCL-*sspA*	This work
AW23-13	AW23 carrying pBR-GP-ω, pACTR-AP-Zif and pCL-*sspA*	This work
AW23-14	AW23 carrying pBR-*mglA*-ω, pACTR-AP-Zif and pCL-*sspA*	This work
AW23-15	AW23 carrying pBR-GP-ω, pACTR-*fevR*-Zif and pCL-*sspA*	This work
AW24	AW20 conjugated with KDZifΔZ; Str^r^, Km^r^	This work
AW24-1	AW24 carrying pBR-*mglA*-ω and pACTR-*sspA*-Zif	This work
AW24-2	AW24 carrying pBR-GP-ω and pACTR-AP-Zif	This work
AW24-3	AW24 carrying pBR-*mglA*-ω, pACTR-*fevR*-Zif and pCL-*sspA*	This work
AW24-4	AW24 carrying pBR-GP-ω, pACTR-AP-Zif and pCL-*sspA*	This work
AW24-5	AW24 carrying pBR-*mglA*-ω, pACTR-AP-Zif and pCL-*sspA*	This work
AW24-6	AW24 carrying pBR-GP-ω, pACTR-*fevR*-Zif and pCL-*sspA*	This work
AW26	AW21 conjugated with KDZifΔZ; Str^r^, Km^r^	This work
AW26-1	AW26 carrying pBR-*mglA*-ω and pACTR-*sspA*-Zif	This work
AW26-2	AW26 carrying pBR-GP-ω and pACTR-AP-Zif	This work
AW26-3	AW26 carrying pBR-*mglA*-ω, pACTR-*fevR*-Zif and pCL-*sspA*	This work
AW26-4	AW26 carrying pBR-GP-ω, pACTR-AP-Zif and pCL-*sspA*	This work
AW26-5	AW26 carrying pBR-*mglA*-ω, pACTR-AP-Zif and pCL-*sspA*	This work
AW26-6	AW26 carrying pBR-GP-ω, pACTR-*fevR*-Zif and pCL-*sspA*	This work
**Plasmids**		
pKD46	λ red expression vector, thermosensitive-30°C; Amp^r^	[47]
pKD4	Plasmid used as the source of the kanamycin resistance marker; Km^r^	[47]
pCP20	Helper plasmid, FLP recombinase, thermosensitive-30°C; Amp^r^, Cm^r^	[47]
pBRGP-ω	Plasmid used to create fusions to the N-terminus of the ω subunit of *E. coli* RNA polymerase; Car^r^, Amp^r^	[32]
pBR-*mglA*-ω	pBRGP-ω carrying *mglA* gene from *F. tularensis* SCHU S4 at *Nde*I and *Not*I sites	[21]
pACTR-AP-Zif	Plasmid used to create fusions to the N-terminus of the Zif protein; Tet^r^	[32]
pACTR-*sspA*-Zif	pACTR-AP-Zif carrying *sspA* gene from *F. tularensis* SCHU S4 at *Nde*I and *Not*I sites	[21]
pACTR-*fevR*-Zif	pACTR-AP-Zif carrying *fevR* gene from *F. tularensis* LVS at *Nde*I and *Not*I sites	[19]
pCL-*sspA*	Modified pCL1920 carrying *sspA* gene from *F. tularensis* LVS; Spec^r^	[19]
pKK214	Low-copy expression vector with the *groEL* promoter of *F. tularensis* LVS; Tet^r^	[55]
p-AB	pKK214 carrying *mglAB* genes from *F. tularensis* SCHU S4 at *Pst*I and *EcoR*I sites	This work
pHis-AB	p-AB carrying His-tagged *mglAB* genes from *F. tularensis* SCHU S4	This work
pSAB	pHis-AB carrying *sspA* gene from *F. tularensis* SCHU S4 at *Spe*I and *Pst*I sites	This work

Str^r^, Km^r^, Amp^r^, Cm^r^, Tet^r^, and Spec^r^ indicate resistant to streptomycin, kanamycin, ampicillin, chloramphenicol, tetracycline, and spectinomycin, respectively.

Y is the amino acid tyrosine.

* the Km gene is inserted in the upstream region.

*E. coli* strains AW23, AW24, and AW26 were used as the reporter strains for the bacterial two-hybrid experiments. The strains were constructed as follows. For the AW23 strain, an *E. coli sspA* knockout mutant (AW18) was constructed in strain FW102 [46] using the protocol described by Datsenko and Wanner, (2000) [47] and reported in Wrench *et al.* (2013) [21]. For AW24, the AW18 strain was used to delete the *relA* gene to obtain AW19, which was then used to delete the *spoT* gene, resulting in the AW20 strain. To construct the AW26 strain, AW18 was used to disrupt the *ppKppX* operon, resulting in the AW21 strain. The reporter strain KDZifΔZ harbors an F’ episome containing the *lac* promoter derivative p*lac*Zif1-61, driving expression of a linked *lacZ* reporter gene [32]. KDZifΔZ was used as donor for conjugation (previously described in [48]) of the recombinant F’ plasmid into the strains AW18, AW20, and AW21, resulting in the reporter strains AW23, AW24, and AW26.

### DNA Manipulations and Gene Cloning

Standard methods were used for chromosomal DNA isolation, restriction enzyme digestion, agarose gel electrophoresis, ligation, and transformation [49]. Plasmids were isolated using spin miniprep kits (Qiagen, Valencia, CA) and PCR products were purified using QIAquick purification kits (Qiagen). All primers used are described in Table 2. The plasmids for protein purification and the bacterial two-hybrid system are described in Wrench *et al.* (2013) [21].

**Table 2:** Oligonucleotides used in this study.

Primer	Sequence (5′→3′)
**qRT-PCR**	
* iglA*-Fw	aatgtccttagcaaacgatgc
* iglA*-Rv	cttttgattttgaggcacca
* pdpD*-Fw	atctgccccaacactaccag
* pdpD*-Rv	gctcagcaggattttgatttg
* ppK*-Fw	gagaaatcaatggtattttcaac
* ppK*-Rv	cttgctggctaactgaga
* ppX*-Fw	gcttgtctatgttggagctag
* ppX*-Rv	agccgctgctgcatgaaaattag
* rpsD*-Fw	tgtcgaagctagcagaagaaa
* rpsD*-Rv	gccagcttttacttgagcaga
IP-*pdpD*P-Fw	aaatcgttgatatcttgatccatac
IP-*pdpD*P-Rv	gcaaccggagcaaaaagtag
IP-*iglA*P-Fw	catcaaccttgaatttgggatt
IP-*iglA*P-Rv	agcaactgtaccagctagagga
IP-*fevR*P-Fw	taaagctactaagctgaaataattgct
IP-*fevR*P-Rv	tcaaaatttccagaatattgattcg
IP-*ppK*P-Fw	cattgctgccgccatattac
IP-*ppK*P-Rv	tgcctttattgcttgaggcta
**Chromosomal Genes Disruption**	
* rela*-Fw	atggttgcggtaagaagtgcacatatcaataaggctggtgaatttgatcc**gtgtaggctggagctgcttcg** b
* relA*-Rv	ctaactcccgtgcaaccgacgcgcgtcgataacatccggcacctggttga**catatgaatatcctccttag** b
* spoT*-Fw	ttgtatctgtttgaaagcctgaatcaactgattcaaacctacctgccgga**gtgtaggctggagctgcttcg** b
* spoT*-Rv	ttaatttcggtttcgggtgactttaatcacgtctggcatcacgcggattt**catatgaatatcctccttag** b
* ppKppX*-Fw	atgggtcaggaaaagctatacatcgaaaaagagctcagttggttatcgtt**gtgtaggctggagctgcttcg** b
* ppKppX*-Rv	ttaagcggcgatttctggtgtactttcttcttcaattttcaaccgccagc**catatgaatatcctccttag** b
**pKK214 Vector Cloning**	
*mglAB*P-*Pst*I-Fw	ggagcgctgcaggtgtatttatgaaaaaaggtactg
*mglABP*-*EcoR*I-Rv	gcggaattcttaatctaacgaaagaacgttatc
*sspAP-Spe*I-Fw	cggactagtctgccggtgatactggaaat
*sspAP-Pst*I-Rv	cgcactgcagcaagcctaccttttgccaga
**Site directed mutagenesis**	
*mglA*His-Fw	ctaggaggatacaatcatg**catcatcatcatcatcat**ttgcttttatacac^c^
*mglA*His-Rv	cgctatagatatcatctttttttgtgtataaaagcaa**atgatgatgatgatgatg**cat

a Underlines indicate the restriction sites.

b Bold indicates the priming site.

c Bold and underline indicate the His-tag.

For the bridge two-hybrid system, one protein is fused to the ω subunit of the *E. coli* RNAP in the pBR-GP-ω plasmid, a second protein is fused to the Zif protein in the pACTR-AP-Zif plasmid, and a third protein is expressed from a replicating modified pCL1920 plasmid [19]. The *F. tularensis* SCHU S4 *mglA* gene was amplified by PCR, and fused to the ω subunit of the RNAP by cloning in the *Nde*I and *Not*I sites of the pBRGP-ω plasmid, to obtain pBR-*mglA*-ω. Plasmids pACTR-*fevR*-Zif (from strain *F. holarctica* LVS) and pCL-*sspA* (*F. holarctica* LVS) were provided by Dr. Simon Dove (Harvard University). The plasmids were co-transformed into the reporter strains (AW23, AW24, and AW26) by natural competence (Table 1).

Plasmids used for immunoprecipitation assays were constructed by first amplifying the *F. tularensis* SCHU S4 *mglAB* genes by PCR, including its native promoter. The PCR product was digested with *Pst*I and *EcoR*I restriction enzymes and ligated to the *Pst*I-*EcoR*I digested pKK214 plasmid [55], to obtain pKK214-P*mglAB* (p-AB). A 6xHis-Tag was added to the N-terminus of *mglA* by site directed mutagenesis, resulting in the plasmid pKK214His-*mglAB* (pHis-AB) (Table 2). The *sspA* gene (including its native promoter) was amplified, digested with *Spe*I and *Eco*RI restriction enzymes and ligated to *Spe*I-*EcoR*I digested pHis-AB, resulting in pKK214-*sspA*-His-*mglAB* (pSAB). Recombinant clones were obtained in *E. coli* DH5α, confirmed by sequencing, and transformed into *F. novicida* strains by cryotransformation.

Cryotransformation was performed as described previously by Mohapatra *et al*. (2007) [50]. Briefly, *F. novicida* strains (WT, Δ*mglA*, Δ*sspA,* Δ*ppK* and Δ*ppX*) were grown to an OD_600_ of 0.5, cells were harvested and resuspended in transformation buffer (10 mM MOPS [morpholinepropanesulfonic acid], 75 mM CaCl_2_, 10 mM RbCl_2_, and 15% glycerol, with pH adjusted to 6.5 with 1 N KOH). One µg of plasmid was mixed with the cells in buffer, incubated for 30 min on ice and flash frozen in liquid nitrogen for 5 min. Cells were thawed at room temperature, plated onto CHOC II plates with tetracycline (10 µg/ml) for WT strain, or CHOC II plates with tetracycline (10 µg/ml) and kanamycin (15 µg/ml) for the mutant strains. Plates were incubated at 37°C for 24 hours. All of the strains are listed in Table 1.

### Protein Purification

Protein purification was performed as previously described [51]. Briefly, the His-tagged fusion proteins in p15TV-*mglA* and pCDF-*sspA* were overexpressed in *E. coli* BL21-Rosetta(DE3) (Novagen). The cells were grown in LB broth at 30°C to an OD_600_ of ∼0.6 and expression was induced with 1 mM isopropyl 1-thio-β-D-galactopyranoside (IPTG). After induction, the cells were incubated at 17°C for 16 h. The cells were harvested, resuspended in binding buffer (500 mM NaCl, 5% glycerol, 50 mM Tris, (pH 8.0), 5 mM imidazole), and stored at −80°C. Cell disruption was carried out using a French press, after the addition of 1 mM phenylmethylsulfonyl fluoride. The lysates were clarified by centrifugation (30 min at 17,000×*g*) and applied to a metal chelate affinity column charged with Ni^2+^. The column was washed (in binding buffer with 15 mM imidazole) and the proteins were eluted from the column in elution buffer (binding buffer with 250 mM imidazole). The purified proteins were dialyzed against 10 mM Tris (pH 8.0), 500 mM NaCl, 2.5% glycerol and kept at −80°C.

### β-galactosidase Assays

Cells were grown at 37°C in LB broth with aeration (200 rpm). Gene expression of the fusion proteins in the AW23-1 strain was induced by the addition of 0.5 mM lactose, at an OD_600_ of 0.2. Culture samples were taken every 30 min, permeabilized with 0.15% sodium dodecyl sulfate (SDS) and 1.5% chloroform in Z-buffer (60 mM Na_2_HPO_4_·7H_2_O, 40 mM NaH_2_PO_4_·H_2_O, 10 mM KCl, 1 mM MgSO_4_, 50 mM β-mercapthoethanol) and assayed for β-galactosidase activity by following the catalytic hydrolysis of the chlorophenol red-β-D-galactopyranoside (CRPG) substrate. Absorbance at 570 nm was read continuously using a Synergy HT 96-well plate reader (Bio-Tek Instruments Inc., Winooski, VT). β-galactosidase activity is expressed in arbitrary units (AU) [52]. Assays were performed in duplicates at least three times. The basal AU was determined from assays performed with the empty plasmids pACTR-AP-Zif+pBR-GP-ω or pBR-GP-ω+pACTR-AP-Zif+pCL-*sspA*. For ease of presentation, the basal AU has already been subtracted from pBR-*mglA*-ω+pACTR-*sspA*-Zif or pBR-*mglA*-ω+pACTR-*fevR*-Zif+pCL-*sspA*, respectively.

### RNA Isolation and Quantitative RT-PCR

*F. novicida* was cultured in modified TSB broth and cells were collected by centrifugation during exponential phase. Total RNA was isolated with a RiboPure™ Bacteria kit (Ambion, Austin, TX) in accordance with the manufacturer’s protocol. cDNAs were synthesized with the Superscript™ first-strand synthesis kit (Invitrogen) in accordance with the manufacturer’s instructions, and stored at −80°C prior to use. Quantitative RT-PCR was carried out in the iCycler, IQ device (Bio-Rad) using Platinum® SYBR® Green qPCR SuperMix for iCycler (Invitrogen) in accordance with the manufacturer’s recommended protocol. The genes *ppK* and *ppX* were then quantified (Table 2). The *rpsD* gene was used as the internal control.

### Polyphosphate Measurements

PolyP was measured using DAPI (4′,6-diamidino-2-phenylindole) as previously described [7]. Briefly, cells suspensions from exponential phase grown cells were washed twice in in 100 mM Tris HCl (pH 7.5) buffer and resuspended in the same buffer to an OD_600_ of 0.2. DAPI was added to a final concentration of 10 µM. After incubation at 37°C for 5 min with agitation, the DAPI fluorescence spectra (excitation, 420 nm; emission, 445 nm) were recorded in a Synergy HT 96-well plate reader (Bio-Tek Instruments Inc., Winooski, VT). The fluorescence was normalized to the optical density of the cells. A standard curve constructed using sodium polyphosphate (Aldrich 305553) was used to calculate the concentration of polyP in the cell samples. The determination of polyP bound to proteins was normalized to protein concentration. The statistical significance of changes in polyP concentration (between the wild type strain and mutant strains) was conducted using a one-tailed Student *t* test.

### Cross-linking and Immunoprecipitation Assays

The procedure described by Laub *et al.,* (2002) [27] was used with some modifications. Ten milliliters of the different strains tested (Fig. 4) were grown in modified TSB broth at 37°C to an optical density of 1 at 600 nm. One hundred microliters of 1 M sodium phosphate, pH 7.6, and 300 µl of 37% formaldehyde were added, and the culture was incubated for 15 min at room temperature with occasional shaking. Glycine (250 mM) was then added to stop the crosslinking during 15 min. Cells were pelleted and washed twice with 10 ml of PBS buffer, pH 7.4. Cells were resuspended in PBS and 1 mM PMSF (freshly prepared) was added. Samples were then sonicated with a Branson sonifier at power 5 for 5 min in 30-sec pulses (DNA sheared to an average size of 0.5–1.0 kbp). After centrifugation, 75 µl of the supernatant containing the sheared DNA was saved as the “total DNA” sample and kept on ice during IP of the rest of the sample. One microgram of monoclonal anti-His serum was added to 1 ml of cross-linked sonicated DNA. After shaking gently at 4°C overnight, 25 µl of a 50% slurry protein Dynabeads Protein A (equilibrated in PBS buffer) was added (Invitrogen). Samples were shaken gently at 4°C for an additional 1 h. Beads were collected by magnet separation and washed five times with PBS buffer and one time with TE buffer. To the total DNA sample, 5 µl of 10% SDS was added. Dynabeads from immunoprecipitated samples were resuspended in 50 µl of TE buffer, pH8. Then all samples were incubated at 65°C overnight to reverse crosslinks. Samples were put in the magnet to remove beads. Twenty microliters of each sample was purified by using a Qiagen (Chatsworth, CA) PCR purification kit, resulting in a final volume of 50 µl of purified total or immunoprecipitated DNA. The DNA in the immunoprecipitates was analyzed using real time quantitative PCR. Primer sequences are listed in Table 2. The entire procedure was carried out three times, and the results were averaged. The standard deviation was less than 10%. The enrichment factor for a given gene was calculated as the ratio of amplified immunoprecipitated DNA in the strain carrying the pSAB plasmid compared to the empty plasmid in the same strain. The statistical significance was determined using a one-tailed Student *t* test.

### Size Exclusion Chromatography

Size exclusion chromatography was performed using 100 µl protein samples. Aliquots contained 24 µM Ft-MglA/Ft-SspA complex and where indicated, 100 µM polyphosphate, prepared in 10 mM Tris (pH 8), 500 mM NaCl. Following 30 min of incubation on ice, samples were injected onto a prepacked Superose 12 10/300 GL (GE Healthcare, Sweden) gel filtration column connected to a LCC-501 plus (Pharmacia Biotech Inc., Piscataway, NJ) equilibrated with 10 mM Tris (pH 8.0), 500 mM NaCl. Filtration was carried out at 4°C, using a flow rate of 0.5 ml/min. The eluted proteins were monitored continuously for absorbance at 280 nm using a UV-M II monitor (Pharmacia Biotech Inc.). Blue dextran 2000 was used to determine the void volume of the column. A mixture of protein molecular weight standards, containing IgG (150 kDa), BSA (66 kDa), Albumin (45 kDa), Trypsinogen (24 kDa), Cytochrome C (12.4 kDa), and Vitamin B12 (1.36 kDa) was also applied to the column under similar conditions. The elution volume and molecular mass of each protein standard was then used to generate a standard curve from which the molecular weight of eluted proteins was determined.

### Differential Scanning Calorimetry (DSC)

DSC measurements were carried using a MicroCal VP-DSC differential scanning microcalorimeter (MicroCal LLC, Northampton, MA). Protein samples were extensively dialyzed against a buffer with 10 mM phosphate (pH 7.9), 500 mM NaCl. Polyphosphate solutions were prepared in dialysis buffer. Prior to loading, all samples were degassed for 30 min at 4°C using a ThermoVac degassing station (MicroCal). Fresh dialysis buffer was used in the reference cell. Samples treated with polyphosphate (100 µM) were incubated at 4°C for 30 min, prior to DSC analysis. Polyphosphate was also added to the reference buffer at equal concentrations. A scan rate of 45°C h^−1^ was used for all experiments, with constant pressure (25 psi) applied to both cells throughout each run. Buffer scans, recorded in the presence or absence of polyphosphate, were subtracted from the corresponding thermograms before analysis. Data was analyzed using the Origin software supplied by the manufacturer (MicroCal). Curves were fit to the data using the non-two-state transition model.

### Isothermal Titration Calorimetry (ITC)

ITC measurements were performed on a VP-Microcalorimeter (MicroCal, Northampton, MA) at 18°C. The protein was thoroughly dialyzed against 10 mM Tris (pH 8.0), 150 mM NaCl. A solution of polyphosphate (100 µM) was directly prepared in dialysis buffer. Each titration involved a series of 5 µl injections of polyphosphate into the protein solution. The mean enthalpies measured from injection of the ligand into the buffer were subtracted from raw titration data before data analysis with ORIGIN software (MicroCal). Titration curves were fitted by a nonlinear least squares method to a function for the binding of a ligand to a macromolecule [53]. From the curve thus fitted, the parameters Δ*H* (reaction enthalpy), *KA* (binding constant, *KA = *1/*KD*), and *n* (reaction stoichiometry) were determined. From the values of *KA* and Δ*H*, the changes in free energy (Δ*G*) and in entropy (Δ*S*) were calculated with the equation: Δ*G* = −*RT* ln*KA* = Δ*H*−*T*Δ*S*, where *R* is the universal molar gas constant and *T* is the absolute temperature.

## Supporting Information

Figure S1 **Basal β-galactosidase activity expression of the strains AW23 (square), AW24 (circle), and AW26 (triangle) carrying the pBR-GP-ω and pACTR-AP-Zif plasmids.** The β-galactosidase activity (expressed in arbitrary units, AU) was determined as described in material and methods.
(TIF)

Figure S2 **Transcriptional activation of** ***lacZ*** **mediated by the interaction between FevR with the MglA/SspA complex.** Different combinations of empty vectors and fused proteins were transformed in the *E. coli* reporter strains: A) AW23 (Δ*sspA*), B) AW24 (Δ*sspA* Δ*relA* Δ*spoT*) and C) AW26 (Δ*sspA* Δ*ppKppX*). The plasmid constructs tested were: pBR-GP-ω+pACTR-AP-Zif+pCL-*sspA* (square: W+Zif+SspA), pBR-*mglA*-ω+pACTR-*fevR*-Zif+pCL-*sspA* (circle: MglA-W+FevR-Zif+SspA), pBR-*mglA*-ω+pACTR-AP-Zif+pCL-*sspA* (diamond: MglA-W+Zif+SspA), pBR-GP-ω+pACTR-*fevR*-Zif+pCL-*sspA* (triangle: W+FevR-Zif+SspA). The β-galactosidase activity (expressed in arbitrary units, AU) was determined as described in material and methods.
(TIF)

Figure S3 **Plasmid pSAB restores growth defect phenotype in** ***F. novicida*** **mutant strains** ***mglA*** **and** ***sspA*** **.** Growth experiments were performed with *F. novicida* WT, Δ*mglA* and Δ*sspA* strains carrying the empty pKK214 plasmid and pSAB. The OD_600_ was recorded at different time points. The strains assayed were WT pKK214 (empty squares), WT pSAB (empty circle), Δ*mglA* pKK214 (pentagon), Δ*mglA* pSAB (left triangle), Δ*sspA* pKK214 (triangle), and Δ*sspA* pSAB (diamond).
(TIF)
