# Supplementary figures and images for: MglA/SspA Complex Interactions Are Modulated by Inorganic Polyphosphate
Source: PLoS One. 2013 Oct 8;8(10):e76428. doi: 10.1371/journal.pone.0076428 (PMC3792966; doi:10.1371/journal.pone.0076428)

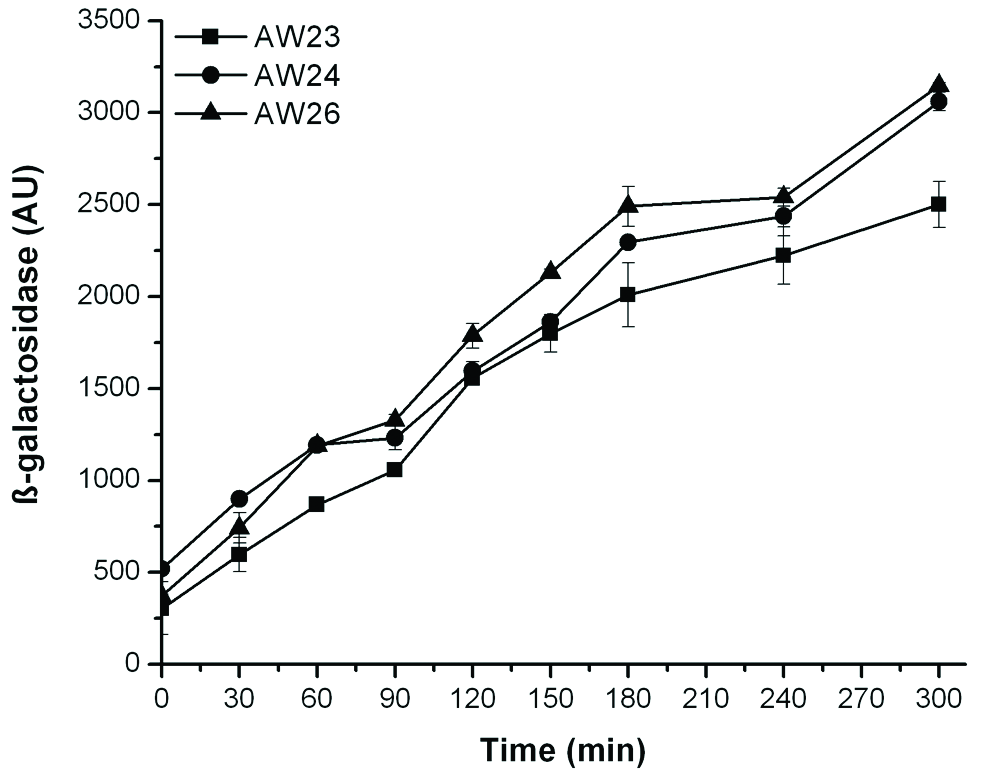

Supplement: Figure S1 — Basal β-galactosidase activity expression of the strains AW23 (square), AW24 (circle), and AW26 (triangle) carrying the pBR-GP-ω and pACTR-AP-Zif plasmids. The β-galactosidase activity (expressed in arbitrary units, AU) was determined as described in material and methods. (TIF) [file pone.0076428.s001.tif]

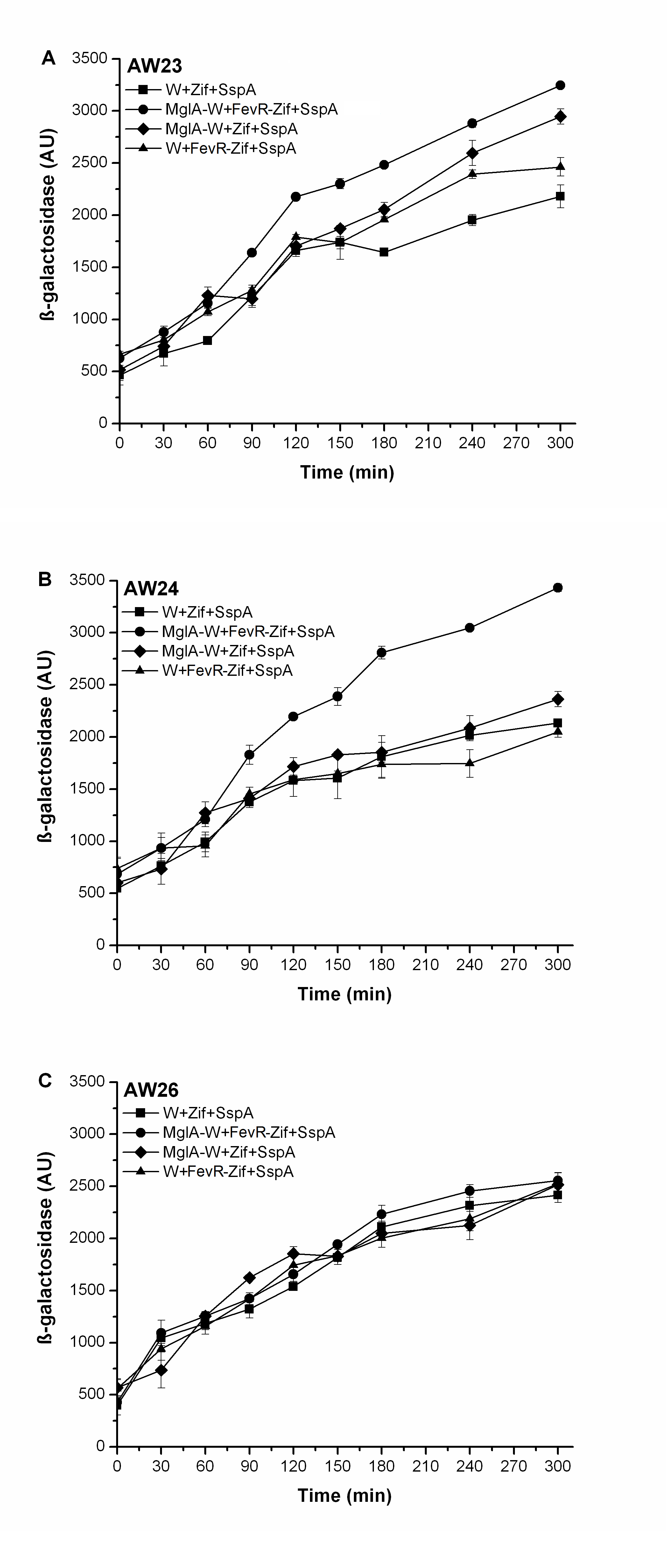

Supplement: Figure S2 — Transcriptional activation of lacZ mediated by the interaction between FevR with the MglA/SspA complex. Different combinations of empty vectors and fused proteins were transformed in the E. coli reporter strains: A) AW23 (ΔsspA), B) AW24 (ΔsspA ΔrelA ΔspoT) and C) AW26 (ΔsspA ΔppKppX). The plasmid constructs tested were: pBR-GP-ω+pACTR-AP-Zif+pCL-sspA (square: W+Zif+SspA), pBR-mglA-ω+pACTR-fevR-Zif+pCL-sspA (circle: MglA-W+FevR-Zif+SspA), pBR-mglA-ω+pACTR-AP-Zif+pCL-sspA (diamond: MglA-W+Zif+SspA), pBR-GP-ω+pACTR-fevR-Zif+pCL-sspA (triangle: W+FevR-Zif+SspA). The β-galactosidase activity (expressed in arbitrary units, AU) was determined as described in material and methods. (TIF) [file pone.0076428.s002.tif]
